# Report on the Cases of Typhoid Fever, or the Common Continued Fever of New England, Which Occurred in the Massachusetts General Hospital, from the Opening of That Institution, in September, 1821, to the End of 1835

**Published:** 1838-08-29

**Authors:** 


*Report on the Cases of Typhoid Fever, or the Common Continued Fever of New England, which occurred in the Massachusetts General Hospital, from the opening of that Institution, in September*, 1821, *to the end of* 1835. *By* James Jackson, M. D., late Attending Physician. 8vo., pp. 95. Boston, 1838.

Dr. Jackson has but rarely appeared before the public in the character of an author, preferring, as he does, to earn silently and tranquilly the honour which awaits the persevering and faithful practitioner, to the questionable glory of weaving theories, or building up imperfect systems, by the premature generalization of a few facts. During the long period of his connection with a large public hospital, a connection which has now continued for seventeen years, he has scarce ventured to produce any work of more formal character than an occasional contribution to a public journal, or a memoir to enrich the transactions of the State Medical Society, of which he is a prominent and respected member. With these exceptions, the results of his experience have been communicated, almost exclusively, in an oral form to the students, who have attended his lectures at the Boston Medical College, or have followed him in his tour of clinical duty. To the truly practical character of the instruction thus given, and to the peculiarly unassuming manner in which it is conveyed, we can bear abundant testimony. Conscientious to an extreme in the statement of facts, and cautious almost to a fault of drawing unwarranted conclusions, he has ever been listened to with that respect and confidence, which nothing short of a pure and ardent love of truth can inspire, and which have rarely fallen to the lot of the most brilliant and eloquent lecturers. It is as a clinical teacher that Dr. J. has especially distinguished himself. The duties of the hospital to which he was and is attached, have ever been performed with a fidelity and completeness which are, perhaps, without a parallel in this or any country. The history of every case, both previous to its admission, and during its stay in the hospital, has been entered, at his dictation, on the records of the house, where each case appears under its appropriate head with the phenomena and prescription of each successive day. The labour necessary to effect this in a hospital containing thirty to forty medical beds was not trifling; and, accordingly, three or four or even five hours of daily attendance in the wards of a public institution was never deemed too much by this practitioner, even when engaged in an extensive and lucrative private practice. This labour, formerly followed up for six months of each year, and lately for four, has furnished to Dr. J. a familiarity with the facts of the cases, which renders him an invaluable clinical instructor, and he has spared no pains in rendering the knowledge, thus acquired, available to his students. A peculiar diffidence and self-mistrust, however, combined with the cautious habits of mind already alluded to, have hitherto prevented him from giving the results of his labours to the public in any systematic form; and it may be doubted whether, in this form, the experience of this enlightened practitioner in disease, is ever destined to be presented to the profession.

The work before us, announced with the modest title of a report, is, we presume, published by the learned society to which it was communicated. It is based on the author's observations at the hospital during fourteen years; almost all the cases referred to, however, having occurred between 1824 and 1835. The number of these which were well marked and decided in character, was three hundred and three; besides sixty-five more doubtful, but two-thirds of which Dr. J. thinks may be added to the former number. Of the three hundred and three decided cases, forty-two proved fatal; of the sixty-five others, but one; so that if forty of the latter be admitted into the account, the mortality will appear to have been forty-three in three hundred and forty-three, or one in eight nearly. Dr. J. assigns as his reason for the adoption of the term typhoid, the identity of the disease with that described by Louis under this title. To the specific grounds of this opinion, we shall presently revert. Among the introductory remarks, we have some curious and valuable information in regard to the mode of practice adopted, both by the author and by the New England practitioners generally, in continued fever. An emetic or cathartic, or both, frequently both in combination, are administered at the outset; then antimonials are given in nauseating doses for twenty-four, thirty-six, or forty-eight hours; and, after this, calomel is resorted to, usually so as to produce slight constitutional affection, but in grave cases pushed to salivation. Local bleeding is very commonly practised, but venesection much less frequently resorted to than in other parts of the country. Dr. J.'s personal experience has tended to lessen his confidence in the calomel and antimonials, but to confirm him in the propriety of early evacuations.

Among the more interesting of the individual cases, is one related, (p. 44,) in which profound stupor continued for three successive days, but without preventing a favourable termination.

It is worthy of observation, that while the disease, in its leading features, remained true to itself, particular symptoms predominated at different periods. For example, epistaxis occurred in one fourth of all the cases. But in 1834, it showed itself in seven of twelve, and, in the same year, in ten of fourteen successive cases; that is, in nearly two-thirds. Deafness occurred in about one-seventh of the whole. In 1833, it was present in seven of eight successive cases. In 1829, of twenty-five cases, twenty-four in succession terminated favourably. Dr. J. finds the facts he has collected unfavourable to the doctrine of contagion.

In a large proportion of the fatal cases, examinations were made after death. But it was not till October, 1833, that the intestines were properly examined. When examined, the diseased appearances in Peyer's glands, described by Louis, were noticed. In all the examinations, eleven in number, from October, 1833, to the end of 1835, similar morbid changes were found.

The following facts are presented, (p. 27,) as illustrating the advantage of early admission into the hospital.

Of ninety cases admitted in the first week of the disease, seven, or 1 in 12.85 were fatal. Of one hundred and thirty-nine admitted in the second week, sixteen, or 1 in 8.68. Of forty-six admitted in the third week, ten, or 1 in 4.6. Of twenty-one admitted after the third week, five, or 1 in 4.2 were fatal. Again: in those who recovered, the average day of convalescence was, in those admitted in the first week, 17.42; in the second, 21.21; in the third, 25.52; in the fourth, or later, 43.93.

That these results prove much in favour of the regimen and treatment adopted in the institution, is evident; but the precise degree of this influence, which no one at all acquainted with the author could for a moment suspect him of wishing to exaggerate, is masked to the reader by the absence of information on two points, one of which could be ascertained with some approach to accuracy. These are—1. The degree, in which the probability of a fatal termination on the one hand, and that of recovery on a particular day on the other, were affected on each successive day by the number of days which had already elapsed. 2. The circumstances which, after a certain time, would determine the dismission of cases from private practice, and their transfer to the wards of a hospital. To the latter consideration we do not attach much importance; though it is evident that the cases of mild character, and which promised well, would, ceteris paribus, be more likely to remain under private treatment than others. No desperate case, we presume, was ever admitted to the hospital; but there are grades of danger below this, not difficult to be perceived by a practised eye, and the more unpromising the aspect of the patient after the lapse of eight or ten days, the stronger in many cases would be the disposition of those around him to have him removed to a public institution, provided sufficient confidence was felt that no hazardous experiments were to be tried, and that the body could not be subjected to post-mortem examination without express permission.

The other consideration alluded to, viz., the operation of time itself in modifying the probability of ultimate death or of recovery on a certain day, is of more importance. The calculation, however, at best, would be a complex one, and its elements could only be furnished by a table which should present the day of convalescence in each favourable, and of death in each fatal case. Such a table the author (having other objects in view) has not thought it necessary to furnish; from a remark, however, which occurs, (p. 33,) we infer that convalescence sometimes occurred on the fourth day, and not unfrequently within the first week.

We have already remarked, that the term typhoid, as applied to the continued fever of New England, has been only recently adopted by Dr. Jackson. The same disease has been heretofore known by the name of typhus, bilious, nervous, and continued fever; and the first of these is the term which Dr. J. himself was accustomed to employ. The change, he tells us, is made in compliance with the suggestion of Dr. Gerhard, of this city, who proposes to restrict the term typhus to the disease recently described by him, (vide *American Journal of the Medical Sciences*, Feb. and Aug., 1837,) as having occurred extensively in the Philadelphia hospital. Dr. J. finds that the fever of New England agrees precisely with the typhoid of Louis, and differs in kind as well as degree from the typhus of Dr. J. We propose, in the remainder of this notice, to verify the first of these positions, so far as symptoms are concerned, by a collation of the numerical results obtained at Boston from 1821 to 1835, with those procured by Louis, in Paris, between 1822 and 1827.

*Diarrhœa.*—Jackson reports two hundred and ninety-seven cases, in reference to diarrhœa—in one hundred and sixty-seven of which it was observed, or 1 in 1.77. Louis (Recherches) gives one hundred and twenty-eight cases, in five of which only it appears to have been absolutely wanting; so that it was present in one hundred and twenty-three of one hundred and twenty-eight cases, or 1 in 1.04. Some uncertainty must obviously rest on this branch of the comparison, as the number of daily discharges which may be considered as constituting diarrhœa, is not stated by either observer. By both it is regarded as an unfavourable symptom. L. remarks, that the frequency of the discharge was proportionate to the severity of the other symptoms; and J. finds that this symptom was followed by death in 1 case in 5.21, while its absence preceded a fatal termination in but one of thirteen cases.

*Pain and tenderness of Abdomen, and Meteorism.*—Of the precise proportion of these occurrences to the whole number of cases, Dr. J. has preserved no precise account, but represents them all as *frequent.* According to the observations of M. Louis, pain occurred in a greater or less degree in all except five; that is, in all the cases which proved fatal, and in seventy-three of eighty-eight which terminated favourably. Meteorism occurred in thirty-four of forty-six cases which proved fatal, and in fifty-five of eighty-eight which recovered.

*Tongue.*—J. notes the tongue as coated or furred in nearly all the cases; dry in one of two cases; denuded in 1 of 5.5; and dark in 1 of 6.28. L., on the contrary, found the tongue nearly natural in one-third of the subjects who died, and in 32 of the 88 cases which recovered. In regard to the particular morbid symptoms presented by this organ in the remaining cases, they are described in terms so different from those employed by Dr. J., that it would serve little purpose to recount them. In general, the French pathologist seems to have attached little importance to the condition of the tongue, the morbid states of which he considers as deserving examination only on their own account, and not as indicating a similar state of the mucous membrane of the stomach. (Rech. ii. 106.)

*Dysphagia.*—A difficulty of deglutition, sufficient to notice, occurred to Dr. J. in twenty-one cases, of which four were fatal. M. Louis met with this symptom in ten cases out of forty-six who died, and in thirteen of fifty-seven severe cases which recovered. This symptom, which M. Louis is not willing to regard as ever merely nervous, and independent of lesion, is by no means always in proportion to the extent of this lesion. Dr. Jackson considers it, when existing in a severe form, as implying a high degree of danger.

*Headach.*—This symptom was noticed by Dr. J. in the early period of almost every case. Louis remarked only four exceptions in the forty-six fatal, two in the fifty-seven grave, and three in the eighty-seven favourable cases. The latter remarks its disappearance at the approach of delirium or stupor.

*Somnolence.*—This symptom was observed by Dr. J. in forty-seven cases, or 1 in 6.44; among the fatal cases, in 1 in 3.81; among the favourable, in 1 of 7.15. Although among the unfavourable symptoms, it was not, even when existing in a great degree, uniformly followed by death. To M. Louis it occurred in all the fatal cases except five, or in eight of nine subjects; of the fifty-seven grave cases which recovered, eight escaped it, and of the thirty-one mild cases, nineteen only gave evidence of its presence. The character of the affection, especially when amounting to stupor, is described in almost the same terms by both authors.

*Delirium.*—This symptom occurred to Dr. Jackson in one hundred and eight cases, or 1 in 2.8; that is in 1 of 3.48 of the favourable, and in 1 of 1.27 of the fatal. This statement is not to be regarded as including the mildest cases. M. Louis found it in thirty-eight of his forty-six fatal cases, but as in two of these it was transitory, and in two an immediate precursor of death, the number may be reduced to thirty-four, or 1 in 1.35. Of the severe cases, delirium was present in thirty-nine out of fifty-six; and of the milder ones, three only manifested it. On the whole, then, delirium occurred in forty-two of eighty-seven who recovered, or in the ratio of one to two nearly.

*Subsultus tendinum.*—This was noted by Dr. J. in 1 case in 8.18, viz., 1 in 3.81 of the fatal, and one in ten of the favourable. By Louis, in four of forty-six fatal, and in three of eighty-eight favourable cases. More importance, however, seems to have been attached to this particular form of spasmodic action, and probably more attention was directed to it at Boston than in Paris.

*Rigidity of Limbs* was noticed by Dr. J. in five of the fatal cases, or 1 in 8.4; and in one of the two hundred and sixty-one favourable; by Louis in four of forty-six of the fatal cases. Cramp was noticed by Louis in one case, and catalepsy by Dr. J., in three.

*Deafness* was noticed by Dr. J., in forty-five cases, or 1 in 6.7, viz., 1 in 4.66 of the fatal, and 1 in 7.25 of the favourable. By Louis, in twenty of thirty fatal, and in thirty-eight of fifty-seven favourable, being precisely the same proportion in both. It is probable that slighter degrees of the affection were recorded by the latter than the former observer.

*Taches roses.*—Of one hundred and six cases noticed by Dr. J. in reference to this symptom, two in three had rose spots, viz., nine of eighteen fatal, and sixty-one of eighty-eight favourable cases. M. Louis found them in twenty-six of thirty-five fatal, and in all the favourable cases, except three, two of which, it is remarked, did not come under treatment till the fourteenth day.

*Sudamina.*—Of the one hundred and six cases noticed by Dr. J., in reference to these vesicles, forty-one were found to exhibit them, or 1 in 2.58. Of the eighteen fatal cases, four had sudamina; of the eighty-eight favourable cases, thirty-seven. Hence Dr. J. finds them a more favourable symptom than rose taches. M. Louis found them in the proportion of two to three of the number examined, both in the fatal and favourable cases.

It would appear from this parallel, and likewise from a comparison of the more general symptoms, that the French disease differed from the American in its greater intensity and fatality, while the main features of both were identical. It would lead us too far to inquire why, in three hundred and three patients, Dr. Jackson should have lost but forty two; while of one hundred and twenty-eight cases treated by Louis, forty proved fatal. It is evident, however, that a peculiar mortality awaited that numerous class of persons who had just exchanged the pure air and regular habits of the country, for the exhalations of the Paris streets, and for the temptations to excess offered by a city life. No class resembling this, is to be found in the capital of New England. E. G. D.

